# Abf1 Is an Essential Protein That Participates in Cell Cycle Progression and Subtelomeric Silencing in *Candida glabrata*

**DOI:** 10.3390/jof7121005

**Published:** 2021-11-25

**Authors:** Grecia Hernández-Hernández, Laura A. Vera-Salazar, Leonardo Castanedo, Eunice López-Fuentes, Guadalupe Gutiérrez-Escobedo, Alejandro De Las Peñas, Irene Castaño

**Affiliations:** 1 División de Biología Molecular, Instituto Potosino de Investigación Científica y Tecnológica (IPICYT), Camino a la Presa San José No. 2055 Col. Lomas 4 Sección, San Luis Potosí CP 78233, Mexico; grecia.hernandez@ipicyt.edu.mx (G.H.-H.); laura.vera@ipicyt.edu.mx (L.A.V.-S.); maria.gutierrez@ipicyt.edu.mx (G.G.-E.); cano@ipicyt.edu.mx (A.D.L.P.); 2 Department of Plant Physiology, Faculty of Biology and Biotechnology, Ruhr University Bochum, Universitätsstrasse, 150 ND3/30, D-44801 Bochum, Germany; castanedo.leonardo@gmail.com; 3 Division of Hematology and Oncology, Department of Pediatrics, University of California, San Francisco, CA 94158, USA; eunice.lopezfuentes@ucsf.edu

**Keywords:** *Candida glabrata*, Abf1, DNA replication, cell cycle, subtelomeric silencing

## Abstract

Accurate DNA replication and segregation is key to reproduction and cell viability in all organisms. Autonomously replicating sequence-binding factor 1 (Abf1) is a multifunctional protein that has essential roles in replication, transcription, and regional silencing in the model yeast *Saccharomyces cerevisiae*. In the opportunistic pathogenic fungus *Candida glabrata*, which is closely related to *S. cerevisiae*, these processes are important for survival within the host, for example, the regulation of transcription of virulence-related genes like those involved in adherence. Here, we describe that *CgABF1* is an essential gene required for cell viability and silencing near the telomeres, where many adhesin-encoding genes reside. *Cg*Abf1 mediated subtelomeric silencing depends on the 43 C-terminal amino acids. We also found that abnormal expression, depletion, or overexpression of Abf1, results in defects in nuclear morphology, nuclear segregation, and transit through the cell cycle. In the absence of *ABF1*, cells are arrested in G2 but start cycling again after 9 h, coinciding with the loss of cell viability and the appearance of cells with higher DNA content. Overexpression of *CgABF1* causes defects in nuclear segregation and cell cycle progression. We suggest that these effects could be due to the deregulation of DNA replication.

## 1. Introduction

Chromatin structure in eukaryotes plays a crucial role in DNA metabolism, such as DNA replication, activation, and repression of transcription, and regional silencing, among others. Each process requires different proteins and the *cis*-acting DNA sequences to which they bind, favoring protein-protein interactions that shape different chromatin structures necessary to trigger specific metabolic functions. In *Saccharomyces cerevisiae* the proteins *Sc*Abf1 and *Sc*Rap1 have been shown to be required for all these processes and, because they perform multiple metabolic functions, they are known as General Regulatory Factors, or GRFs [1,2,3]. *Sc*Abf1 is a sequence-specific DNA-binding protein for which there are many recognition sites (~181) throughout the genome, including promoters of genes activated (50) or repressed (36) by *Sc*Abf1, the silent mating-type loci (*HML* and *HMR*), the region X of many yeast telomeres and sites known as ARS (Autonomous Replication Sequences), which confer stability and the ability to replicate, to plasmids [4,5,6]. Many ARSs indeed function as origins of replication in the chromosomes and are recognized by *Sc*Abf1. One special case is the sequences that function as ARSs present in the silent mating-type *loci*, *HMR* and *HML*. These *loci* are each flanked by *cis*-acting elements called silencers (*E* and *I*) that are essential to mediate regional silencing of the mating information at each locus. In particular, the silencers *HMR-E* and *HML-I* both contain different combinations of three specific DNA sequences recognized by the Origin of Recognition Complex (ORC), *Sc*Rap1, and *Sc*Abf1, respectively. Indeed, these *cis*-elements mediate silencing of the genes present at *HML* and *HMR* and confer plasmid stability as they are functional ARSs, suggesting a connection between initiation of DNA replication and silencing [6,7]. More recently, it has been shown that *Sc*Abf1, along with other GRFs like *Sc*Rap1, regulates the accuracy of transcription initiation by preventing inappropriate initiation and pervasive transcription [8].

The *ABF1* gene encodes *Sc*Abf1, an essential and abundant protein that consists of 731 amino acids. *Sc*Abf1 is composed of two regions involved in DNA-binding (an atypical Zinc-finger domain and a DNA-binding region) and a C-terminal domain that is required for all its known functions: transcriptional repression and activation, DNA replication, and regional silencing at the silent mating-type *loci* [6,9,10].

*Candida glabrata* is a haploid yeast phylogenetically related to *S. cerevisiae*, which has emerged as a frequent and important opportunistic pathogen associated with high mortality rates in intensive care units worldwide [11,12,13]. *C. glabrata* is frequently found as a commensal, tightly adhered to the mucosa of the gastrointestinal or urinary tracts of healthy individuals; however, it can cause severe systemic infections when the mucosal barrier is breached or there is severe immunosuppression of the human host.

The ability to adhere to epithelial or endothelial mucosal tissue of the host is thought to be an important first step for *C. glabrata* infections, and this ability is largely mediated by a family of cell wall proteins called the Epa adhesins encoded by the *EPA* genes, most of which are located at subtelomeric regions [14,15]. The *EPA1* gene encodes the major adhesin (Epa1) and is the only member of the family expressed in vitro in the standard laboratory strain BG14 [16,17]. Expression of *EPA1* is negatively controlled by several layers of negative regulation, including subtelomeric silencing that spreads from the telomere through up to 20 Kb [18,19,20], promoter-specific repression by a *cis*-acting negative element (NE) downstream from *EPA1* [21], and repression by the formation of a chromatin loop between a subtelomeric, *cis*-acting protosilencer (Sil2126) and sequences flanking the *EPA1* ORF [22,23]. We have shown that the *C. glabrata* ortholog of *ScABF1* (CAGL0J01177g) is required for the formation of the chromatin loop that represses *EPA1* along with other silencing proteins that participate in subtelomeric silencing such as *Cg*Sir2, *Cg*Sir3, and *Cg*Sir4 (forming the SIR complex), and *Cg*Rap1 [23]. 

However, up to date, *CgABF1* has not been characterized; it is not known whether it also participates in general subtelomeric silencing, DNA replication or if it is an essential gene in *C. glabrata*. Here we show that the *CgABF1* gene is essential for cell viability, that *Cg*Abf1 participates in subtelomeric silencing through the C-terminal domain, and is also required for correct DNA replication and normal transit through the cell cycle. We suggest that these are all processes that are likely important for the ability of *C. glabrata* to persist in its human host.

## 2. Materials and Methods

### 2.1. Strains, Plasmids, and Primers

All strains, plasmids, and oligonucleotides used are listed in Supplementary Tables S1–S3, respectively. 

### 2.2. Media

Yeast was grown in standard yeast media as described previously with 2% agar added for plates [24]. Synthetic complete (SC) medium contains 1.7 g/L yeast nutrient base (YNB) without (NH_4_)_2_SO_4_ or amino acids, 5 g/L (NH_4_)_2_SO_4_ and supplemented with 0.6% casamino acids (CAA) and 2% glucose. When needed, Nourseothricin (Gold Biotechnology^®^. St. Louis Missouri, USA) was added to liquid YPD media at a concentration of 50 µg/mL. Minimal media is YNB without (NH_4_)_2_SO_4_ or amino acids; 2% glucose and 5 g/L (NH_4_)_2_SO_4_ as nitrogen source. For the selection of Nourseothricin resistance (Nat^R^) in minimal media, ammonium sulfate was substituted for 1 g/L of monosodium glutamate. For the plasmids with the shut-off expression from the *MET3* promoter, ON media (expression of *MET3* promoter) is minimal media as described above or with 1 g/L monosodium glutamate instead of ammonium sulfate. For the OFF media 0.2 mM of each methionine (met) and cysteine (cys) were added to the ON media. To prepare 5-fluoroorotic acid (5-FOA; Toronto Research Chemicals, Toronto, Ontario, Canada) media, 0.9 g of 5-FOA and 25 mg of uracil were added to 1 L of SC medium. 5-FOA compound is toxic to cells that express the *URA3* gene. 

Yeast extract-peptone-dextrose (YPD) medium contains 10 g/L yeast extract, 20 g/L peptone, and supplemented with 2% glucose. When required, YPD plates were supplemented with nourseothricin (AG Scientific, San Diego, CA, USA) at 100 µg/mL and at 50 µg/mL for liquid YPD media. Bacteria were grown in LB medium as described previously [25]. LB medium contained 5 g/L yeast extract, 10 g/L tryptone, 5 g/L NaCl. All plasmid constructs were introduced into strain DH10 by electroporation, and 100 µg/mL carbenicillin (AG Scientific, San Diego, CA, USA) was added to select for plasmids. For plates, 1.5% agar was used. Both bacteria and yeast were grown at 30 °C.

### 2.3. Yeast Transformation

Yeast transformations with digested or supercoiled plasmids were performed as previously described using the LiOAc/salmon sperm carrier DNA/PEG method [26,27].

### 2.4. Growth Assays in Liquid and Solid Media

Cells were grown to stationary phase for 48 h in YPD, CAA, or YNB. To determine the duplication time, cells of each strain were grown in the appropriate liquid media. Stationary phase cultures were adjusted to an OD_600_ of 0.01 in the corresponding media and 300 µL dispensed in a 100 well plate. Growth was automatically recorded using a Bioscreen C analyser at 30 °C (Oy, Growth Curves) with constant shaking and OD measurements taken every 15 min during a period of 48 h. The doubling time of each strain was calculated as described elsewhere [28]. Growth curves in liquid media were performed 3 separate times and with technical triplicates each repetition. The data shown represents the mean of the three experiments.

For solid media experiments, stationary phase cultures were adjusted to an OD_600_ of 1.0 with sterile water, and 10-fold serial dilutions were made in a 96-well plate. Five microliters of each dilution were spotted on to YPD plates. For temperature sensitivity assay, a total of 5 µL of each dilution was spotted onto YPD, then incubated at 30, 37 or 45 °C, and photographed every 24 h during three days. All solid media experiments were done in triplicate, and photographs of representative experiments are presented.

### 2.5. Construction of Null Mutant and Truncated Allele of the ABF1 Gene

Since *ScABF1* is essential for cell viability in *S. cerevisiae*, we designed a strategy to determine whether *CgABF1* is an essential gene in *C. glabrata*. To do this, we first constructed a replicative plasmid containing the full-length ORF of *CgABF1* plus the 5′ and 3′ flanking regions of this gene (1 Kb upstream and 800 bp downstream, primers #1589 and #1881, Supplementary Table S3), and the *URA3* selection marker (pCI12). We also generated a disruption plasmid for *CgABF1*. Briefly, the 5′ (primers #1589 and #1881) and 3′ (primers #1561 and #1562, Table S3) flanking intergenic regions of the gene to be deleted were PCR amplified and cloned into pYC44 integrative plasmid [29] on either side of the nourseothricin expression cassette (conserving the relative orientation of the chromosomal locus of *ABF1*) (pCI42). The plasmid generated was used to replace the wild-type *CgABF1* gene with the nourseothricin cassette by homologous recombination in a one-step gene replacement procedure to generate *abf1*∆::Nat^R^ allele. For this, we first transformed the parental strain BG14 (Supplementary Table S1) with the plasmid containing the wild-type *CgABF1* gene and *URA3* marker (pCI12). This strain was then transformed with the linearized fragment from the knock-out plasmid (pCI42). The plasmid was previously digested with enzymes that cut within both fragments of the cloned 5′ and 3′ flanking regions, generating homologous ends to the *ABF1* gene in the *C. glabrata* genome. Transformants were selected on plates supplemented with nourseothricin (AG Scientific, San Diego, CA, USA) at 100 µg/mL. Homologous recombination and allele replacement of the gene was verified by PCR analysis using primers annealing within the nourseothricin cassette and outside of the cloned 5′ and 3′ flanking regions. We also verified the absence of the gene deleted by the inability to amplify an internal fragment from *ABF1* by PCR. 

To construct the C-terminal truncated allele of *ABF1* (that lacks the last 43 amino acids that correspond to part of the CS2 domain important for silencing in *S. cerevisiae* [10], we designed primers to amplify the truncated ORF of *ABF1* ending at amino acid 436 using primers #1559 and #1880 (Supplementary Table S3). The fragment obtained was cloned into the integrative vector pYC44, generating plasmid pCI30. A 714 bp fragment containing the 3′ UTR region of *ABF1* was cloned into pCI30 flanking the nourseothricin cassette (pCI32) [23]. Plasmid pCI32 was digested with *Bsg*I within the region of *C. glabrata* homology and transformed into the parental BG14 selecting for Nat^R^. 

### 2.6. Plasmid Loss Assay to Determine If ABF1 Is Essential in C. glabrata

We used the null mutant described above, which contains a *URA3* plasmid carrying *ABF1* (pCI12) and a deletion of the chromosomal copy of *ABF1* (*abf1∆*). This strain, as well as the BG14 parental strain transformed with pCI12, and BG14 with empty vector, were grown at 30 °C for 48 h in YPD and diluted into fresh media and grown for another 24 h, as described previously [28]. This was repeated three times. Ten-fold serial dilutions were plated on YPD plates for viable counts on SC + 5-FOA plates to select for the loss of the *URA3* plasmid. Ura^+^ cells die on SC + 5-FOA plates; therefore, only cells that have lost the *URA3* plasmid can grow on SC + 5-FOA. The percentage of cells without plasmid was calculated by counting the number of colonies on SC + 5-FOA divided by the number of colonies on YPD (viable count). The results shown are the mean of three biological replicates ± SD. The statistical analysis used was One-Way ANOVA using GraphPad.

### 2.7. Plasmid for Tagging Abf1 with Flag or c-Myc under the Repressible MET3 and Inducible MT-1 Promoters

We generated a replicative vector to tag *Cg*Abf1 at the N-terminal end with Flag or c-Myc epitopes followed by a linker (5 repetitions of GA) and under the control of the promoters of either the *MET3* gene (METhionine requiring), which is repressed by methionine and cysteine or *MT-1* gene (MetalloThionenin 1) which is inducible with CuSO_4_.

For the construction of the plasmid with the Flag tag under the *MET3* promoter, we digested the plasmid pGH5 that contains the full length of *ABF1* flanked by *Cla*I restriction sites; the released fragment was cloned into the plasmid pRM153 [30], which contains the P*_MET3_* promoter followed by the Flag tag and the GA linker to generate the N-terminal translational fusion (pVS1: pP*_MET3_*::Flag-*ABF1*-T*_HIS3_*.Nat^R^).

We amplified the full-length *ABF1* gene with primers #2353 and #2354 (Supplementary Table S3), both of which contain *Cla*I restriction sites, to clone it into the plasmid pGH3 [23], which contains the P*_MT1_* promoter fusion with the c-Myc tag and the GA linker to generate translational fusions at the N-terminal end of the protein of interest. The final plasmid (pGH8: pP*_MT-1_*::c-Myc-*ABF1*-T*_HIS3_*.Nat^R^) [23] was transformed into *E. coli*, and after verifying that the gene fragment was cloned in the correct orientation, it was transformed into *C. glabrata* (see yeast transformation section). All final plasmids were sequenced to confirm that constructs were correct and had no mutations.

### 2.8. Growth Assays with the Repressible Promoter MET3 in C. glabrata

We generated a collection of mutants that contain the plasmid pVS1 (pP*_MET3_*::Flag-*ABF1*-T*_HIS3_*. Nat^R^), the *MET3* promoter is repressible by the addition of cysteine and methionine [31]. We conducted a series of growth assays in order to determine the minimum concentration needed to achieve the shutdown of the promoter (Supplementary Table S4). Then we conducted the experiments with two different media: ON medium (*MET3* promoter is active), which is YNB (no methionine or cysteine), where the ammonium sulfate ((NH_4_)_2_SO_4_) was replaced with sodium glutamate (C_5_H_8_NO_4_Na) (1 g/L) when Nat had to be added for selection of the plasmids; OFF medium (no expression of the *MET3* promoter) is the same as ON media, but adding 0.2 mM of L-cysteine (cys) and L-methionine (met). In SC (which contains casaminoacids) or YPD media, the *MET3* promoter was also fully repressed. 

### 2.9. Viability Assays

Stationary phase cultures (48 h in ON media) of strain *abf1Δ*/pVS1 (pP*_MET3_*::Flag-*ABF1*.Nat) were diluted to OD_600_ 0.1 in either ON (YNB) or OFF (YNB plus 0.2 mM each met and cys) and incubated at 30 °C with shaking. Samples were taken every hour for 6 h in one experiment and from 7 to 12 h in a second experiment, and 24 h samples were also taken from both experiments. Cell suspensions from the samples were serially diluted in sterile water, and the appropriate dilutions were plated on ON (YNB) media. Colonies were counted, and CFUs were calculated per mL of cell suspension. All experiments were performed in triplicate, and the data shown corresponds to the mean of the three independent experiments ± SD. Statistical analyses used were Two-Way ANOVA and Tukey’s test using GraphPad, PRISM.

### 2.10. Reporter URA3 Gene Expression Assays (5-FOA Sensitivity Assays)

We generated a collection of mutants that contain the *URA3* reporter gene inserted near different telomeres. These strains allow us to measure silencing at different positions in the selected subtelomeric regions, as shown in Figure 7 and in Supplementary Figure S4. A plate growth assay was used to determine the level of silencing as described previously [18,19]. Strains were grown in YPD for 48 h to stationary phase and then were adjusted to an OD_600_ of 1.0 in sterile water, and 10-fold serial dilutions were made in 96-well plates. Subsequently, ~5 µL of each dilution was spotted onto YPD, SC (no uracil), and SC + uracil + 5-FOA plates (with or without CuSO_4_ 50 µM for the induction of P*_MT-1_* containing plasmids), with the replica-plating tool (Frogger) (NUNC™). The plates were incubated for 48 h at 30 °C and then photographed.

### 2.11. Cell Cycle Progression Experiments by FACS

Strains to be analyzed were grown to stationary phase in the appropriate media and then inoculated in either ON or OFF media at an OD_600_ 0.5 and incubated with shaking at 30 °C. Samples were taken every 3 h and stained with Sytox Green as follows. Cells were centrifuged and 1 × 10^7^ cells were resuspended in 450 µL of sterile water. One-thousand and fifty microliters of 100% ethanol were slowly added and gently mixed, after which cells were incubated from 1–12 h at 4 °C. Cells were collected by centrifugation and washed with 1 mL 50 mM Tris pH 8.0. The cell pellet was resuspended in 500 µL of RNAse (2 mg/mL) for 2 h at 37 °C after which cells were centrifuged and resuspended in 200 µL of proteinase K (5 mg/mL) for 20 to 45 min at 37 °C. Cells were centrifuged and washed with 1 mL 50 mM Tris pH 8.0. Cells were resuspended in 50 µL of 50 mM Tris pH 8.0 and stained with SYTOX^TM^ Green (5 mM, Thermo Fisher Sientific, Watham, MA, USA: 1 µL SYTOX^TM^ Green in 1 mL 50 mM Tris pH 8.0). Cells were analyzed by flow cytometry (FACS) using BD FACSCalibur Flow Cytometer with Cell Quest Pro software, and the data were analyzed with FlowJo^TM^.

### 2.12. Fluorescence Microscopy

Strains to be analyzed were grown to stationary phase in the appropriate media and then inoculated in either ON, OFF, with 25 μM CuSO_4_ or without CuSO_4_ media at an OD_600_ 0.5 and incubated with shaking at 30 °C. Samples were taken every 3 h until 9 h. 1 mL samples of each strain were centrifuged and washed with PBS, and then fixed with ethanol 70% for 1 h. Cells were washed with PBS and stained with DAPI for nuclei visualization. Cells were analyzed with Axio Imager.m2 Microscope (Carl Zeiss). Images were analyzed with the software AxioVision v. 4.8.2.0 image browser. At least 100 cells were counted for each strain and condition except in the presence of copper, where 65 and 49 cells at 6 h and 9 h were counted, respectively. Representative images are shown.

## 3. Results

### 3.1. Candida glabrata Encodes the ABF1 Gene

**Figure 1:**
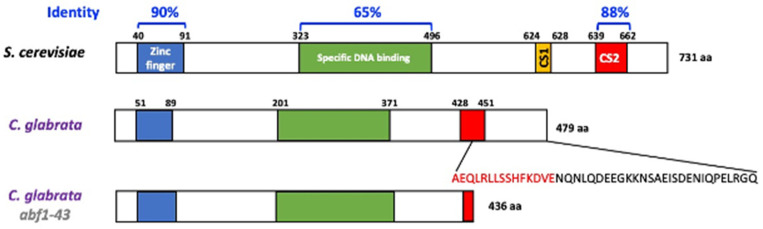
*C. glabrata* encodes the *Cg*Abf1 protein with conserved DNA-binding and regulatory domains. Schematic representation of Abf1 from *S. cerevisiae* and *C. glabrata*, *Cg*Abf1 has 479 aa and *Sc*Abf1 has 731 aa. The overall identity between *Sc*Abf1 and *Cg*Abf1 across the entire length of the proteins is 63.87%. The blue and green rectangles correspond to the DNA binding domains, the yellow rectangle corresponds to the CS1 domain in *Sc*Abf1 and the red rectangles to the CS2 domain responsible for chromatin remodeling and transcriptional regulation. Alignments were performed using ClustalW with the program McVector. We generated a *CgABF1* truncated allele, *Cgabf1-43*, which lacks the last 43 amino acids of *Cg*Abf1, deleting 15 highly conserved residues at the CS2 domain (in red letters) that mediate transcriptional silencing in *S. cerevisiae* (See Materials and Methods). The structure of the *Abf1-43* truncation mutant protein is shown at the bottom (total of 436 amino acids).

The Abf1protein of *S. cerevisiae* is involved in several important processes, including, among others, DNA replication, transcriptional regulation (activation and repression), and silencing of the silent mating loci *HMR* and *HML* [5,6,7,10]. The *C. glabrata* genome harbors the orthologous *CgABF1* (CAGL0J01177g) gene, which encodes a predicted protein of 479 amino acids with 63.87% overall identity with *ScABF1* (Figure 1). Similar to *Sc*Abf1, *Cg*Abf1 is predicted to contain a bipartite DNA-binding domain: Zn finger domain (residues 51–89) and a specific DNA-binding domain (residues 201–371) sharing 90 and 65% identity with the corresponding domains of *Sc*Abf1 (Figure 1). *Cg*Abf1 also contains a conserved C-terminal activation domain called CS2 (residues 428–451) that is 88% identical to the *Sc*Abf1 CS2 domain, which in *S. cerevisiae* is involved in chromatin remodeling and transcriptional regulation [5,10] (Figure 1 and Supplementary Figure S1). When deletions are made in the C-terminal end of the *S. cerevisiae* protein, silencing and other functions are impaired. We generated a C-terminal deletion allele (*abf1-43*) that lacks the last 43 amino acids, which have been shown to be necessary and sufficient for silencing at the *HMR-E locus* in *S. cerevisiae* [10]. This deletion removes the last 15 amino acids from the CS2 domain to the end of the protein (28 additional amino acids for a total of 43 removed) (Figure 1).

### 3.2. Abf1 Is Essential for Cell Growth in C. glabrata

To determine whether *Cg*Abf1 is essential for cell viability in *C. glabrata*, we designed two different experiments: (1) a plasmid loss assay and (2) a conditional expression system (“shut off plasmid”) using the repressible promoter from the *MET3* gene (P*_MET3_*) (see Materials and Methods).

**Figure 2:**
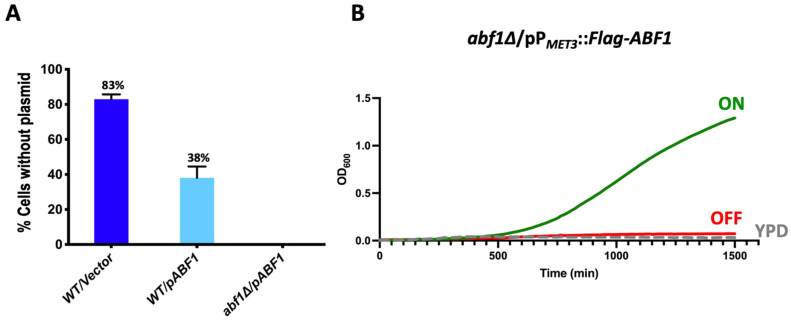
*Cg*Abf1 is an essential protein for growth in *C. glabrata*. (**A**) The indicated strains were grown in YPD rich media (without selection) for 72 h, diluting in fresh media every 12 h after which cells were diluted and plated on YPD and incubated 48 h at 30 °C. Plates were replica-printed onto SC + 5-FOA, SC—uracil and YPD plates to determine the number of Ura^−^ colonies. The graph shows the percentage of cells that lost the plasmid in each culture, and the numbers represent the mean of three separate experiments ± SD. (**B**) Cells from strain *abf1∆*/pP*_MET3_*::Flag-*ABF1* (shut off plasmid) were grown in YNB minimal media (ON) for 48 h. Cells were adjusted to an OD_600_ of 0.01 and inoculated into three media: ON (in green) (YNB minimal media); OFF (in red) (YNB + 0.2 mM each cys and met), and YPD rich media (in broken gray lines). The OD_600_ was recorded every 15 min in Bioscreen C equipment. The graph represents the mean of three biological replicates with three technical replicates each (see Section 2.4 and Section 2.6).

For the plasmid loss assay, we first introduced a replicative plasmid in the parental strain (BG14) with the *ABF1* gene under the control of its own promoter and the counter selectable marker *URA3* (pCI12). In this strain, we deleted the native *ABF1* gene by homologous recombination using a knock-out plasmid (pCI45), containing the Nat^R^ cassette flanked by the intergenic 5′ and 3′ regions of *ABF1* (Supplementary Figure S2A). In a second step, we eliminated the Nat^R^ cassette generating strain CGM3584 (Supplementary Table S1). If *ABF1* is essential for viability in *C. glabrata*, we expected that the strain *abf1∆*/p*ABF1*.*URA3* would not be able to lose the plasmid with the *ABF1* gene. Conversely, if *ABF1* is not essential, this strain would lose the plasmid after repeated cycles of growth in the absence of selective pressure (rich media). To determine the frequency of plasmid loss, we used the plate growth assay on SC + 5-FOA plates since cells expressing *URA3* die in the presence of 5-FOA and only cells that have lost the *URA3* plasmid (Ura^−^ cells) can grow on these plates. Figure 2A shows that the strain *abf1∆*/p*ABF1*.*URA3*, cannot lose the complementing plasmid as measured by the absence of 5-FOA^R^ colonies. This result is in agreement with the fact that *ScABF1* is also essential for viability in *S. cerevisiae* [6,10].

It should be noted that in the parental strain, more than half of the cells (62%) retained an extra copy of *ABF1* gene driven by its own promoter (the endogenous and the episomal copies), suggesting that a higher level of transcription of *ABF1* may be advantageous under these conditions.

For the “shut off plasmid” in which we cloned the full-length *ABF1* gene fused with the Flag epitope at the N-terminal end and driven by the repressible promoter from the *MET3* gene (pVS1: pP*_MET3_*::Flag-*ABF1*). This plasmid was transformed into the *abf1∆*/p*ABF1*.*URA3* strain followed by plasmid shuffling (Materials and Methods and Supplementary Figure S2A). The resulting strain (*abf1∆*/pP*_MET3_*::Flag-*ABF1*.Nat^R^) allows us to use a conditional expression system where *ABF1* is expressed in minimal media without amino acids and completely repress the expression of *ABF1* when methionine and cysteine are added to the media. Figure 2B shows the growth of the null *ABF1* mutant strain with the repressible *ABF1* plasmid (*abf1∆*/pP*_MET3_*::Flag-*ABF1*.Nat^R^) in three different media: YPD (rich media), ON (minimal media without amino acids), and OFF (minimal media supplemented with met and cys). If *ABF1* is essential for growth in *C. glabrata* as suggested by the inability to lose the *ABF1* plasmid in the *abf1∆* strain, we expected that repressing the P*_MET3_* promoter would prevent the growth of this strain. Figure 2B shows that this strain only grew in ON medium but not in the OFF or YPD media, both of which contain amino acids, indicating that *ABF1* is essential for cell growth in *C. glabrata*.

### 3.3. Repression of ABF1 Results in Loss of Cell Viability

**Figure 3:**
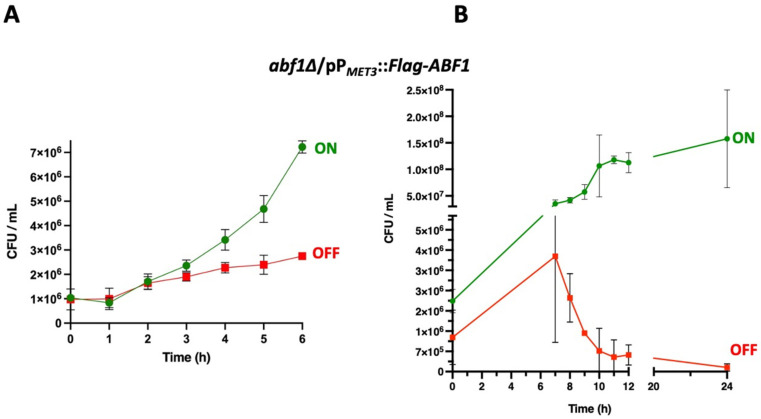
Repression of *ABF1* results in loss of cell viability. (**A**) Stationary phase cultures of strain *abf1∆*/pP_*MET3*_::Flag-*ABF1* were adjusted to an OD_600_ of 0.1 and diluted in either YNB minimal media (ON in green) or in YNB + 0.2 mM each cys and met (OFF in red) and incubated at 30 °C with shaking. Samples were taken to determine viable counts (expressed as colony-forming units or CFU) every hour for the first six hours. (**B**) In an independent set of experiments, samples were taken to determine CFUs as in section A, every hour starting at 7 h after inoculation into each medium until 12 h and another sample at 24 h (graph on the right). All the results shown represent the mean of three independent experiments, and bars correspond to the SD. Analysis of the data was done with Two-Way ANOVA and Tukey’s test using GraphPad, PRISM (See Section 2.9).

We used the *ABF1* shut-off plasmid to determine whether the absence of Abf1 causes cell death. To do this, we grew the *abf1∆* strain containing the plasmid with Flag-*ABF1* fusion under the control of the *MET3* promoter in minimal media in the absence of amino acids (ON media). We then added methionine and cysteine to repress the promoter and took aliquots every 60 min for 12 h and then at 24 h, to determine cell viability. As shown in Figure 3A, during the first 6 h of repression of *ABF1* in the *abf1∆*/pP*_MET3_*::Flag-*ABF1* strain, there is only a two-fold increase in the number of colony forming units (CFU), but after 8 h of repression, viability starts decreasing, and after 24 h, there is a ±10-fold drop (Figure 3B). However, the cells incubated in ON media continue replicating (Figure 3A,B and Supplementary Table S5).

### 3.4. Overexpression of ABF1 Is Toxic in C. glabrata

**Figure 4:**
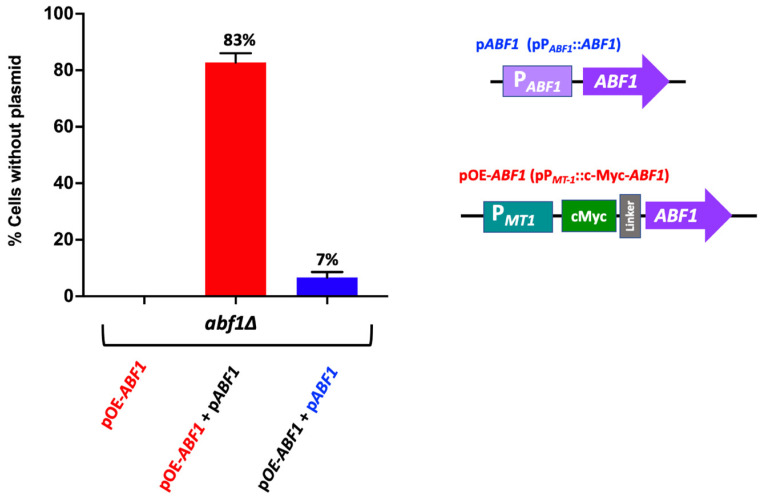
Over expression of *ABF1* is toxic in *C. glabrata*. Schematic representation of the plasmids transformed in the *abf1∆* strain. The first plasmid contains the full-length *ABF1* gene driven by its own promoter (p*ABF1*+) and a second plasmid with the amino-terminal tagged version (c-Myc) of *ABF1* driven by the overexpressing promoter from *MT-1* gene (pOE-*ABF1*). These plasmids were transformed simultaneously, and the loss of each plasmid was determined after passages every 12 h for 72 h in rich media YPD (without selection). The graph shows the percentage of cells that lost the corresponding plasmid (the mean of three independent experiments ± SD, see in Section 2.6). The red bar indicates loss of the over-expressing plasmid pc-Myc-*ABF1* (OE-*ABF1*); the blue bar indicates loss of the plasmid p*ABF1*.

To determine whether overexpression of Abf1 results in toxicity in *C. glabrata* as has been reported for *S. cerevisiae* [32,33], we generated an epitope-tagged version of Abf1 by constructing a plasmid that contains a translational fusion of the c-Myc tag at the 5′ end of *ABF1*, separated by a linker, and under the control of the copper inducible promoter P*_MT-1_* from the *MT-1* gene (CAGL0D01265g) (pGH8) (Supplementary Figure S2B). This construct complements the *abf1∆* strain as determined by the ability to exchange the pP*_ABF1_*::*ABF1*.*URA3* plasmid for the plasmid carrying the c-Myc-*ABF1* tagged version in the *abf1∆* background (Supplementary Figure S2). We also showed that the c-Myc tagged Abf1 (pGH8) is functional for silencing activity mediated by the protosilencer Sil2126 when the Sil-reporter system is placed 32 kb away from telomere E-R (See López-Fuentes et al. [23]. The *abf1∆* strain carrying both plasmids lost the plasmid with the inducible promoter at a much higher frequency (83% of cells lost the pP*_MT1_*::c-Myc-*ABF1* plasmid), than the plasmid where *ABF1* is driven by its own promoter (pP*_ABF1_*::*ABF1*) under non-inducing conditions (Figure 4).

We also determined the growth rate of the parental (*ABF1*) and the *abf1∆* strains in the presence of the overexpressing plasmid P*_MT-1_*::c-Myc-*ABF1* under inducing (with 50 µM CuSO_4_) or non-inducing conditions (without CuSO_4_) in different media SC, YNB or YPD. Table 1 shows that overexpression of *ABF1* by induction with copper in the medium, decreases the growth rate both, in the parental strain (duplication time from 66 min to 89 under inducing conditions), as well as in the strain carrying the C-terminal truncation of the last 43 amino acids (*abf1-43*) where the duplication time increased from 70 to 106 min under the induction of the *MT-1* promoter.

### 3.5. Misexpression of Abf1 Results in Cells with Aberrant Nucleus Morphology and Segregation and Delayed Passage through the Cell Cycle in C. glabrata

**Figure 5:**
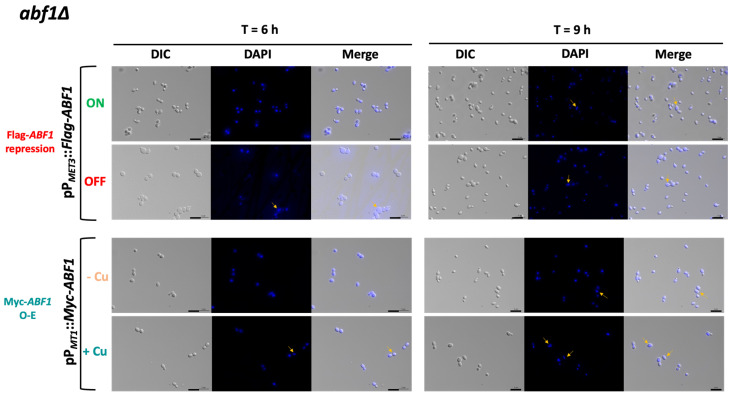
*Cg*Abf1 is required for nuclear integrity. Cells were grown to stationary phase in ON media; then, the OD_600_ was adjusted to 0.5 and incubated at 30 °C in the following media depending on the strain: ON (in green, YNB minimal media) or OFF (in red, YNB plus 0.2 mM each cys and met) or in—Cu (in orange, YNB minimal media) or +Cu (cyan, YNB + 25 µM CuSO_4_) as indicated. Samples were taken at 3, 6, and 9 h of incubation, stained with DAPI, and visualized and photographed under an Axio Imager.m2 fluorescence microscope. Images of the 6 and 9 h are shown. Yellow arrows point to cells with nuclei defects. From left to right, DIC (differential interference contrast), DAPI (blue), Merge (DIC + DAPI). Scale bar corresponds to 5 μm.

Since *ScABF1* is required for DNA synthesis, we asked whether deprivation or overexpression of *ABF1* results in changes in nuclear morphology or cell cycle defects. To do this, we first used the *abf1∆* strain with the *ABF1* repression plasmid (pVS1: pP*_MET3_*::Flag-*ABF1*) to determine the nuclear morphology at different time points after the repression of *ABF1*. Figure 5 (top panels) shows that after 6 h of Flag-*ABF1* repression, some cells appear to have a less well-defined nucleus with fuzzier DAPI staining (~16%), and after 9 h of the absence of Flag-*ABF1*, there are some cells with more than one nucleus (2.5%). At both time points, the cells appear larger in the absence of Flag-Abf1 than in ON media, and there were more budding cells (small budded and large budded) compared to the condition where Flag-*ABF1* is expressed (57.5% in OFF vs. 18.5% in ON at 9 h) (Supplementary Table S6), which suggests that cells in OFF media spend more time in the S or S/G2 phases of the cell cycle than cells in ON media. It should be noted that even under conditions where the *MET3* promoter is active, around 1.3% of the cells at 9 h also appear to have more than one nucleus, suggesting that this promoter is not as strong as the *ABF1* native promoter. Furthermore, cells from the wild-type parental strain did not show abnormal nuclear morphology or more than one nucleus.

**Figure 6:**
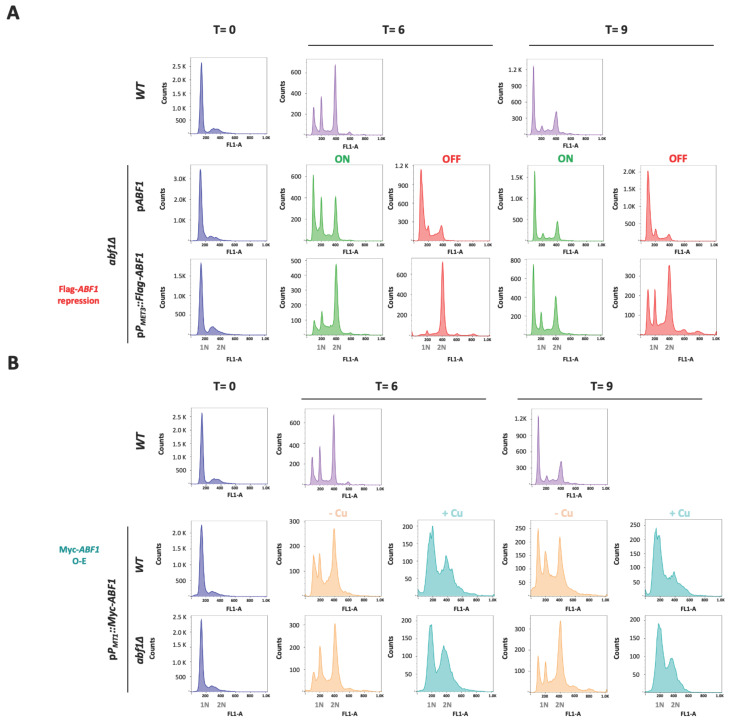
Absence or overexpression of *CgAbf1* causes defects in the cell cycle progression. Stationary phase (SP) cells (T = 0) grown in YNB minimal medium were adjusted to an OD_600_ 0.5 in ON (in green, YNB minimal media); OFF (in red, YNB + 0.2 mM each cys and met),—Cu (Orange, YNB minimal media) or + Cu (teal blue, YNB + 25 μM CuSO_4_) media, depending on the strain, and then incubated 9 h at 30 °C. Samples were taken every three hours and stained with SYTOX™ Green for DNA content analysis by Flow Cytometry (FACS). Representative histograms for DNA content quantification with SYTOX Green are shown; “Y” axis shows the cells count, “X” axis corresponds to the intensity of fluorescence signal from SITYOX Green. 1N and 2N marks in the “X” axis correspond to G1 and G2 DNA content. (**A**) From left to right, T = 0 h (in blue) corresponds to histograms from the indicated strains in SP. T = 6 h histograms show the DNA content after 6 h incubation of: *WT* strain (light purple), *abf1Δ* strain with the native *ABF1* promoter p*P_ABF1_*::*ABF1*, or shut-off plasmid (p*P_MET3_::Flag-ABF1*), in ON (green) or OFF (red) media. T = 9 h are the same as T = 6 h but after 9 h of incubation in each media. (**B**) Same as in A but using the overexpression plasmid as follows. From left to right, T = 0 (blue) histograms show the DNA content of strains from stationary phase cultures; T = 6 correspond to samples after 6 h of *WT* strain (light purple), strains with the overexpression plasmid (p*P_MT1_::Myc-ABF1*) in *WT* and *abf1Δ* backgrounds in—Cu (orange, non-inducing conditions) and + Cu (teal blue, inducing conditions) media. T = 9 same as T = 6 but after 9 h of incubation. To facilitate the comparison with the *WT* strain, these strain’s histograms were duplicated for panels A and B, as all the strains were run together in this experiment and the same *WT* culture served as a control for the rest of the strains and conditions. Experiments were performed three times and the histograms from a representative experiment are shown. Data were analyzed with the software FlowJo.

We also determined the cell cycle progression of the cells in the absence of Flag-*ABF1* compared to the *abf1∆* complemented with the plasmid expressing *ABF1* under its own promoter (pCI12), as well as with the parental strain (*ABF1*). We used stationary phase cells of each strain (T = 0 h) and then diluted each culture into fresh ON or OFF media and took samples after 6 h or 9 h to determine DNA content by flow cytometry. As shown in Figure 6A, 6 h after Flag-*ABF1* repression, cells accumulate in the G2 DNA content phase while the cells in the ON media have started to divide, showing a small proportion of cells with G1 DNA content. After 9 h in OFF media, the cells have apparently bypassed the arrest in G2 and started cycling so that a relatively large proportion of cells with G1 DNA content are detected. In addition, a few cells appear to have higher DNA content equivalent to 3 C and 4 C (visible as small peaks at 600 and 800 fluorescence intensity units in the FL-1A channel, Figure 6A bottom panels), consistent with some observed cells containing two or three nuclei at this time point in the DAPI staining (Figure 5, top panels). In contrast, the cells with the plasmid driving *ABF1* with its own promoter continue cycling albeit at a slower rate since the OFF media causes slow growth as it contains met and cys (Supplementary Table S4).

To test whether over-expression also causes aberrant nuclear morphology and defects in the transit through the cell cycle, we transformed both the parental (*ABF1*) and the *abf1∆* strains with the over-expression plasmid pGH8, where Myc-*ABF1* is induced by adding copper to the medium. Cells from each culture were grown to stationary phase and then diluted into non-inducing (−Cu) and inducing media (+Cu). Figure 5 shows (bottom panels) that overexpression of Myc-*ABF1* in both strains causes the appearance of multiple cells (~16%) with more than one nucleus after 9 h of c-Myc-Abf1 induction, compared to the medium in the absence of copper (~1.25%). In addition, passage through the cell cycle is abnormal, since stationary phase cells (T = 0 h) start cycling 6 h after dilution into media containing 25 µM of CuSO_4_ (Figure 6B); however, there is a large population of cells that have an intermediate DNA content between the characteristic G1 and G2 peaks, which correspond to cells in S-phase, compared to the cells without copper or the wild-type, untransformed strain. After 9 h of overexpression, a population with higher DNA content starts appearing, particularly in the wild-type strain (*WT*) with the pP*MT-1*::Myc-*ABF1* plasmid in which, in addition to the native *ABF1* promoter driving normal levels of expression of *ABF1*, there is additional over-expression of c-Myc-*ABF1* from the induced *MT-1* promoter. This is consistent with the large percentage of budded cells under inducing conditions vs. non-inducing conditions at 6 h (65.1% vs. 35.1%). These effects are most likely due to overexpression of *ABF1* and not an effect caused by CuSO_4_ since the *WT* strain in the presence of this relatively low concentration of copper shows normal duplication time (Table 1) and a very similar pattern of DAPI staining in the absence or presence of copper.

### 3.6. Subtelomeric Silencing at Telomeres E-R and B-L Require the C-Terminal 43 Amino Acids of Abf1

**Figure 7:**
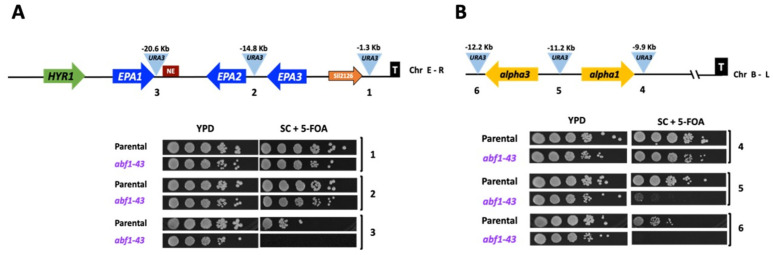
Abf1 plays a role in silencing at different subtelomeric regions in *C. glabrata.* (**A**) Schematic representation of the subtelomeric region of the Chr E-R in *C. glabrata*. The light blue triangles represent the position of three independent insertions of the *URA3* reporter, and the distance from the telomere is indicated above each insertion. The numbers beneath the triangles correspond to the insertions carried by each strain in the plate growth assay below. 5 µL of ten-fold serial dilutions of cells grown to stationary phase were spotted onto the media indicated, incubated at 30 °C for 48 h, and photographed. (**B**) Schematic representation of the subtelomeric region of Chr B-L. Yellow arrows represent *alpha3* and *alpha1* genes at the *MTL3* silent locus and their relative orientation at the Chr B-L. The numbers below the insertions correspond to the numbers in the plate growth assay as in Figure 7A. The experiments were performed three independent times, and photographs from a representative experiment are shown (see in Section 2.10).

*Sc*Abf1 has been shown to play a role in silencing the silent mating-type cassettes (*HML* and *HMR*) in *S. cerevisiae*, and the C-terminal end of *Sc*Abf1 is known to be required for silencing [6,10]. We constructed a truncation mutant in *ABF1*, called *abf1-43*, lacking the last 43 amino acids that eliminate almost all the CS2 conserved region (Figure 1). This mutant is viable but has a longer duplication time compared to the parental strain, and it is also temperature-sensitive (Table 1 and Supplementary Figure S3). To determine whether *Cg*Abf1 is required for silencing, we constructed different strains containing the *URA3* reporter gene integrated at various distances from different telomeres in the wild-type background and in the *abf1-43* mutant. Silencing activity by *abf1-43* was assayed by the ability of the strains carrying each reporter insertion to grow on plates lacking uracil (SC-ura plates) where only cells that express the *URA3* reporter can grow and on plates containing 5-FOA where cells expressing *URA3* convert 5-FOA to a toxic compound and die. As shown in Figure 7A, insertions 1 and 2, where the reporter gene *URA3* is closer to the telomere (1.3 and 14.8 Kb, respectively), were efficiently silenced in the *abf1-43* mutant. However, when the reporter was inserted 20.6 Kb away from the telomere (insertion three), the level of silencing was clearly decreased in the *abf1-43* mutant compared to the parental (*ABF1* strain). In addition, the *abf1-43* strain is also defective in silencing at telomere B-L (where the silent *MTL3* locus is localized) when the *URA3* reporter is inserted at a distance of 11 Kb or more from the telomere (Figure 7B, insertions 5 and 6), although the insertion closest to the telomere is not affected by the absence of the last 43 amino acids of *Cg*Abf1. Instead, at the Chr I-R where *EPA4* and *EPA5* form a 15 Kb inverted repeat, the strong silencing of the reporter *URA3* at different positions in this telomere was as efficient in the parental strain as in the *abf1-43* mutant (Supplementary Figure S4).

## 4. Discussion

The organization of DNA into chromatin and the particular spatial arrangement of the eukaryotic chromatin has a profound impact on the regulation of gene expression and other essential DNA metabolic functions like DNA replication, DNA damage repair, and regional silencing (reviewed in [34]).

In *S. cerevisiae*, *Sc*Abf1 is an essential protein, classified as a GRF since it participates in a variety of important metabolic functions such as transcriptional activation and repression of many genes involved in diverse metabolic pathways, regional silencing, ribosome biogenesis, the establishment of the pre-replicative complex during the cell cycle and DNA repair, as it is part of the nucleotide excision repair subcomplex [5,35,36,37]. *Sc*Abf1 has also been shown to locally alter chromatin structure by generating regions of altered nucleosome occupancy in the vicinity of *Sc*Abf1 binding sites throughout the genome near promoters regulated by *Sc*Abf1 [38]. In addition, *Sc*Abf1 and *Sc*Rap1 prevent ectopic initiation of transcription at promoters by binding and occluding possible inappropriate pre-initiation complex formation [8]. Involvement in all of these processes could explain why *Sc*Abf1 is essential in *S. cerevisiae* [10,39].

*CgABF1* is an essential gene in *C. glabrata.*

The *C. glabrata* ortholog of *ScABF1* (*Cg*Abf1) has conserved the three most important domains for the function of *Sc*Abf1: the Zinc finger domain at the N-terminus of the protein, which is 90% identical with the corresponding domain in *Sc*Abf1, the specific DNA binding domain near the center of the protein (65% identical) and the CS2 domain at the C-terminus (88% identical) (Figure 1). The CS2 domain is required for all the known functions of *Sc*Abf1, including transcriptional regulation, DNA replication, regional silencing, and reorganization of the chromatin structure [10].

In this work, we have determined that, as is the case in *S. cerevisiae*, in *C. glabrata*, *CgABF1* is also required for cell viability since (a) a null *abf1∆* mutant is unable to lose the complementing *CgABF1* plasmid (Figure 2A) and (b) repression of Flag-*CgABF1* from a shut-off plasmid results in the absence of growth and a large drop in viability in the *abf1∆* background (Figure 2B and Supplementary Table S5). Therefore, it is possible that in *C. glabrata, Cg*Abf1 also participates in essential functions such as DNA replication, transcriptional regulation, and regional silencing.

Misexpression of *CgABF1* causes defects in cell cycle progression.

We found that repression of *CgABF1* results in an abnormal cell cycle progression as *abf1∆* cells complemented with the plasmid containing Flag-*ABF1* driven by the repressible promoter from the *MET3* gene, arrest transiently when Flag-*ABF1* is repressed, but then continue cycling, which results in a small peak with DNA content equivalent to 3C and 4C (Figure 6A bottom panels). Indeed, repression of Abf1 results in the appearance of cells with more than one nucleus and also many cells with abnormally stained nuclei that appear as fragmented nuclei (Figure 5, top panels).

On the other hand, the overexpression of *CgABF1* also leads to an abnormal cell cycle, characterized by the initial accumulation of cells in S and G2 phases, after which cells override the partial arrest and continue cycling, resulting in the accumulation of cells in G1 and S phases (Figure 6B). This correlates with a large proportion of budded cells after 6 h (~65%) and the appearance of cells with two and sometimes three nuclei (Figure 5). In addition, we observed a small number of cells that display higher than G2 DNA content (Figure 6B). We also found that overexpression of *CgABF1* significantly increases the duplication time in the parental *ABF1* background as well as in the C-terminal truncation mutant, which removes most of the CS2 conserved region (Table 1). These data are in agreement with the reported toxicity of *ScABF1* overexpression experiments [32,33].

The abnormal DAPI staining and nuclear segregation of either depletion or overexpression of *Cg*Abf1 might explain the drop in cell viability after *Cg*Abf1 repression and point to an important role of *Cg*Abf1 in DNA replication and passage through the cell cycle in *C. glabrata*. These data agree well with the described role of *Sc*Abf1 in DNA replication, whereby studying point mutations in *Sc*Abf1 and mutants in the *cis*-acting ARS binding sites in a plasmid showed that these mutations resulted in plasmid instability [40]. The C-terminal end of the protein is required for DNA replication activity, in particular the CS2 conserved domain [10].

Previous work has shown that the *C. glabrata* cell cycle progression shares many features with the corresponding process in *S. cerevisiae*. There are 253 origins of replication throughout the *C. glabrata* genome and a well-conserved ARS consensus sequence (ACS) [41] as well as high conservation of the functional domains of both Abf1 proteins. This is all consistent with the idea that *Cg*Abf1 participates in DNA replication in *C. glabrata*.

Recently, Shor et al. [42] described that *C. glabrata* cells continue dividing in the presence of DNA damage induced by the addition of methyl methane sulfonate (MMS), which produces a substantial loss of viability. In our experiments, we also observed the absence of a prolonged arrest under repression or overexpression of *CgABF1*, which probably contributes to the large drop in viability after depletion of *Cg*Abf1.

It should be pointed out that the promoter from the *MET3* gene is not strong enough to provide normal growth to the *abf1∆* null strain, since even under conditions where it is expressed (minimal media without amino acids), the cells grow slowly compared to the same strain with the plasmid with *ABF1* driven by its own promoter (127 min vs. 76 min; Supplementary Table S4). Therefore, in *C. glabrata*, *Cg*Abf1 is probably also an abundant protein whose levels are tightly controlled during the cell cycle. Additionally, the lack of proper regulation of the *MET3* promoter could also account for part of the abnormal growth of the *abf1∆* null strain containing this plasmid.

The C-terminal domain of *Cg*Abf1 participates in subtelomeric silencing in *C. glabrata*.

In this work, we found that *Cg*Abf1 participates in subtelomeric silencing in at least two different subtelomeric regions: E-R and B-L, and this function is dependent on the last 43 amino acids of *Cg*Abf1, which include most of the highly conserved CS2 domain (Figure 7). The role *Cg*Abf1 plays in subtelomeric silencing is only detected at distances >11 Kb in B-L telomere and >20 Kb in the case of the subtelomeric E-R region. We have shown previously that in *C. glabrata*, the propagation of the silent chromatin from the telomeres can extend up to >20 Kb (depending on the telomere) [18,19,20]. It is possible that the effect of *Cg*Abf1 in silencing can only be detected at positions sufficiently distant from the respective telomere so that the silencing propagating from the telomere is diminished. At regions closer to the telomere, there is likely redundant silencing activity of all the silencing proteins acting at subtelomeric regions, and the contribution of *Cg*Abf1 to silencing is masked. Consistent with the contribution of *Cg*Abf1 to subtelomeric silencing at the E-R region, we have determined that *Cg*Abf1 and *Cg*Rap1 are recruited throughout this region, where a cluster of the adhesin-encoding genes *EPA1*, *EPA2*, and *EPA3* reside [23].

On the other hand, at telomere I-R where silencing propagates over 23 Kb from the telomere, the effect in the silencing of the *URA3* reporter by *Cg*Abf1-43 is not observed. This is probably because silencing at this telomere is even stronger than at other telomeres since a stem and loop structure may be formed, as *EPA4* and *EPA5* form an inverted repeat, contributing to a strong silencing at this telomere, which would hide the possible contribution of *Cg*Abf1 silencing at this telomere (Supplementary Figure S4).

In this regard, *Cg*Abf1 also conserves the function in the silencing of *Sc*Abf1, where it contributes to regional silencing shown in experiments using an artificial *HMR*-E silencer containing an ARS consensus sequence and two consensus binding sites for Abf1 [43].

One of the proposed mechanisms of *Sc*Abf1 action is that its binding to the B-domain of ARS1 of *S. cerevisiae* prevents nucleosome positioning in the adjacent A domain [44]. In fact, *Sc*Abf1 can compete with histones to generate nucleosome-free regions to favor the access of other proteins and regulate transcription or DNA replication [39].

Therefore, when *Sc*Abf1 binds to the different binding sites throughout the genome, it can change the local chromatin structure [38]. This effect may be driven by regulating nucleosome positioning at the regions where this protein binds. Nucleosome distribution is not random nor determined only by favorable DNA sequences. Instead, the action of different proteins like transcription factors, and general regulatory factors such as Abf1 and Rap1, usually have a strong effect and determine nucleosome positioning [45]. It is possible that *Cg*Abf1 acts similarly to *Sc*Abf1, changing the local chromatin structure by controlling nucleosome positioning in the vicinity of its binding sites near promoters, ARSs, or silencers and protosilencers.

Future experiments will be directed at answering some of these questions.

## Figures and Tables

**Table 1:** Duplication time of strains overexpressing c-Myc-*ABF1* (pP_MT1_::c-Myc-*ABF1*).

Strain ^a^	Type of Promoter Expressing *ABF1* in Plasmid ^b^	Duplication Time (min) ± SD ^c^	
		YPD	YPD + 50 µM CuSO_4_
WT (*ABF1*^+^)	-	59.3 ± 1.2	54.6 ± 0.44
*abf1∆*/pP_*ABF1*::_*ABF1*	*ABF1* wt promoter	56.2 ± 0.1	Nd ^d^
WT/pP_*MT1*_::Myc-*ABF1*	Inducible (overexpressing)	66.1 ± 0.4	89.6 ± 0.4
*abf1-43*	-	72.8 ± 5.9	65.14 ± 1.8
*abf1-43*/pP*_MT1_*::Myc-*ABF1*	Inducible (overexpressing)	70.6 ± 0.6	106.2 ± 1.5

^a^ WT is the parental strain (*ABF1*^+^); *abf1-43* is the strain carrying the deletion of the last 43 amino acids of Abf1 and *abf1∆* is the strain carrying a deletion of *ABF1* in the chromosome rescued by a plasmid expressing *ABF1* from its native promoter. Each strain was also transformed with the plasmid, where an N-terminal epitope-tagged *ABF1* was overexpressed from the copper inducible promoter from the *MT-1* gene. ^b^ Description of the promoter from where the *ABF1* gene was transcribed from the plasmid in each strain. ^c^ Duplication time in min ± standard deviation (SD) of each strain in each media. Non-inducing media (YPD). Inducing conditions is YPD + 50 µM copper sulfate. ^d^ Nd means Not determined. Values represent the mean of three independent biological replicates.

## Data Availability

You might choose to exclude this statement if the study did not report any data.

## Supplementary Materials

The following are available online at https://www.mdpi.com/article/10.3390/jof7121005/s1, Figure S1: *Cg*Abf1 is similar to its ortholog *Sc*Abf1 and conserves the functional domains. Figure S2: Plasmid shuffling scheme to generate a “shut off *ABF1* system” or an “*ABF1* overexpression system” (OE-*ABF1)* in the *abf1∆* background. Figure S3: The *abf1-43* mutant has a longer duplication time and is temperature-sensitive at 45 °C. Figure S4: *Cg*Abf1 is not required for silencing at telomere I-R where a stem-loop structure may be formed. Table S1: *Candida glabrata* and *Escherichia coli* strains used in this study; Table S2: plasmids used in this study; Table S3: oligonucleotides used in this study; Table S4: Duplication times of strains containing *ABF1* driven by the *MET3* promoter; Table S5: Cell viability of strains expressing *ABF1* under the repressible *MET3* promoter; Table S6: Percentage of unbudded and budded cells under repression or overexpression of *ABF1*.

## References

[1] Buchman A.R., Kimmerly W.J., Rine J., Kornberg R.D. (1988). Two DNA-binding factors recognize specific sequences at silencers, upstream activating sequences, autonomously replicating sequences, and telomeres in *Saccharomyces cerevisiae*. Mol. Cell. Biol..

[2] Kimmerly W., Buchman A., Kornberg R., Rine J. (1988). Roles of two DNA-binding factors in replication, segregation and transcriptional repression mediated by a yeast silencer. Embo J..

[3] Yarragudi A., Miyake T., Li R., Morse R.H. (2004). Comparison of ABF1 and RAP1 in Chromatin Opening and Transactivator Potentiation in the Budding Yeast *Saccharomyces cerevisiae*. Mol. Cell. Biol..

[4] Lee T.I., Rinaldi N.J., Robert F., Odom D.T., Bar-Joseph Z., Gerber G.K., Hannett N.M., Harbison C.T., Thompson C.M., Simon I. (2002). Transcriptional Regulatory Networks in *Saccharomyces cerevisiae*. Science.

[5] Miyake T., Reese J., Loch C.M., Auble D.T., Li R. (2004). Genome-wide Analysis of ARS (Autonomously Replicating Sequence) Binding Factor 1 (Abf1p)-mediated Transcriptional Regulation in *Saccharomyces cerevisiae*. J. Biol. Chem..

[6] Rhode P.R., Sweder K.S., Oegema K.F., Campbell J.L. (1989). The gene encoding ARS-binding factor I is essential for the viability of yeast. Genes Dev..

[7] Loo S., Laurenson P., Foss M., Dillin A., Rine J. (1995). Roles of ABF1, NPL3, and YCL54 in silencing in *Saccharomyces cerevisiae*. Genetics.

[8] Challal D., Barucco M., Kubik S., Feuerbach F., Candelli T., Geoffroy H., Benaksas C., Shore D., Libri D. (2018). General Regulatory Factors Control the Fidelity of Transcription by Restricting Non-coding and Ectopic Initiation. Mol. Cell.

[9] Cho G., Kim J., Rho H.M., Jung G. (1995). Structure-function analysis of the DNA binding domain of *Saccharomyces cerevisiae* ABF1. Nucleic Acids Res..

[10] Miyake T., Loch C.M., Li R. (2002). Identification of a Multifunctional Domain in Autonomously Replicating Sequence-Binding Factor 1 Required for Transcriptional Activation, DNA Replication, and Gene Silencing. Mol. Cell. Biol..

[11] Kullberg B.J., Arendrup M.C. (2015). Invasive Candidiasis. N. Engl. J. Med..

[12] Pappas P.G., Lionakis M.S., Arendrup M.C., Ostrosky-Zeichner L., Kullberg B.J. (2018). Invasive candidiasis. Nat. Rev. Dis. Prim..

[13] Toda M., Williams S.R., Berkow E.L., Farley M.M., Harrison L.H., Bonner L., Marceaux K.M., Hollick R., Zhang A.Y., Schaffner W. (2019). Population-Based Active Surveillance for Culture-Confirmed Candidemia—Four Sites, United States, 2012–2016. MMWR. Surveill. Summ..

[14] Xu Z., Green B., Benoit N., Schatz M., Wheelan S., Cormack B. (2020). De novo genome assembly of *Candida glabrata* reveals cell wall protein complement and structure of dispersed tandem repeat arrays. Mol. Microbiol..

[15] Xu Z., Green B., Benoit N., Sobel J.D., Schatz M.C., Wheelan S., Cormack B.P. (2021). Cell wall protein variation, break-induced replication, and subtelomere dynamics in *Candida glabrata*. Mol. Microbiol..

[16] Cormack B.P., Falkow S. (1999). Efficient homologous and illegitimate recombination in the opportunistic yeast pathogen *Candida glabrata*. Genetics.

[17] Cormack B.P., Ghori N., Falkow S. (1999). An Adhesin of the Yeast Pathogen *Candida glabrata* Mediating Adherence to Human Epithelial Cells. Science.

[18] Castano I., Pan S.-J., Zupancic M., Hennequin C., Dujon B., Cormack B.P. (2004). Telomere length control and transcriptional regulation of subtelomeric adhesins in *Candida glabrata*. Mol. Microbiol..

[19] Peñas A.D.L., Pan S.-J., Castaño I., Alder J., Cregg R., Cormack B.P. (2003). Virulence-related surface glycoproteins in the yeast pathogen *Candida glabrata* are encoded in subtelomeric clusters and subject to RAP1- and SIR-dependent transcriptional silencing. Genes Dev..

[20] Rosas-Hernández L.L., Juárez-Reyes A., Arroyo-Helguera O.-E., Penas A.D.L., Pan S.-J., Cormack B.P., Castaño I. (2008). yKu70/yKu80 and Rif1 Regulate Silencing Differentially at Telomeres in *Candida glabrata*. Eukaryot. Cell.

[21] Gallegos-García V., Pan S.-J., Juárez-Cepeda J., Ramírez-Zavaleta C.Y., Martin-Del-Campo M.B., Martínez-Jiménez V., Castano I., Cormack B., Penas A.D.L. (2012). A Novel Downstream Regulatory Element Cooperates with the Silencing Machinery to Repress EPA1 Expression in *Candida glabrata*. Genetics.

[22] Juárez-Reyes A., Ramírez-Zavaleta C.Y., Medina-Sánchez L., Peñas A.D.L., Castaño I. (2012). A Protosilencer of Subtelomeric Gene Expression in *Candida glabrata* with Unique Properties. Genetics.

[23] López-Fuentes E., Hernández-Hernández G., Castanedo L., Gutiérrez-Escobedo G., Oktaba K., Penas A.D.L., Castaño I. (2018). Chromatin Loop Formation Induced by a Subtelomeric Protosilencer Represses EPA Genes in *Candida glabrata*. Genetics.

[24] Sherman F., Fink G.R., Hicks J.B. (1986). Methods in Yeast Genetics.

[25] Ausubel F.R., Brent R.E., Kingston d.D., Moore J.G., Seidman J.A., Struhl K. (2001). Current Protocols in Molecular Biology.

[26] Castaño I., Kaur R., Pan S., Cregg R., Peñas A.D.L., Guo N., Biery M.C., Craig N.L., Cormack B.P. (2003). Tn7-Based Genome-Wide Random Insertional Mutagenesis of *Candida glabrata*. Genome Res..

[27] Gietz R.D. (2014). Yeast transformation by the LiAc/SS carrier DNA/PEG method. Methods Mol. Biol..

[28] Gutiérrez-Escobedo G., Orta-Zavalza E., Castaño I., Peñas A.D.L. (2013). Role of glutathione in the oxidative stress response in the fungal pathogen *Candida glabrata*. Curr. Genet..

[29] Yáñez-Carrillo P., Orta-Zavalza E., Gutiérrez-Escobedo G., Patron-Soberano A., Peñas A.D.L., Castaño I. (2015). Expression vectors for C-terminal fusions with fluorescent proteins and epitope tags in *Candida glabrata*. Fungal Genet. Biol..

[30] Robledo-Márquez K., Gutiérrez-Escobedo G., Yáñez-Carrillo P., Vidal-Aguiar Y., Orta-Zavalza E., Briones-Martín-Del-Campo M., Peñas A.D.L., Calderone R., Castaño I. (2016). *Candida glabrata* encodes a longer variant of the mating type (MAT) alpha2 gene in the mating type-like MTL3 locus, which can form homodimers. FEMS Yeast Res..

[31] Zordan R.E., Ren Y., Pan S.-J., Rotondo G., Penas A.D.L., Iluore J., Cormack B.P. (2013). Expression Plasmids for Use in *Candida glabrata*. G3 Genes Genomes Genet..

[32] Sopko R., Huang D., Preston N., Chua G., Papp B., Kafadar K., Snyder M., Oliver S.G., Cyert M., Hughes T.R. (2006). Mapping Pathways and Phenotypes by Systematic Gene Overexpression. Mol. Cell.

[33] Stevenson L.F., Kennedy B., Harlow E. (2001). A large-scale overexpression screen in *Saccharomyces cerevisiae* identifies previously uncharacterized cell cycle genes. Proc. Natl. Acad. Sci. USA.

[34] Bonev B., Cavalli G. (2016). Organization and function of the 3D genome. Nat. Rev. Genet..

[35] Fermi B., Bosio M.C., Dieci G. (2016). Promoter architecture and transcriptional regulation of Abf1-dependent ribosomal protein genes in*Saccharomyces cerevisiae*. Nucleic Acids Res..

[36] Kawasaki Y., Kojima A., Seki T., Sugino A., Kim H.-D. (2006). Reconstitution of*Saccharomyces cerevisiae*prereplicative complex assemblyin vitro. Genes Cells.

[37] Reed S.H., Akiyama M., Stillman B., Friedberg E.C. (1999). Yeast autonomously replicating sequence binding factor is involved in nucleotide excision repair. Genes Dev..

[38] Ganapathi M., Palumbo M.J., Ansari S.A., He Q., Tsui K., Nislow C., Morse R.H. (2010). Extensive role of the general regulatory factors, Abf1 and Rap1, in determining genome-wide chromatin structure in budding yeast. Nucleic Acids Res..

[39] Yarragudi A., Parfrey L.W., Morse R.H. (2006). Genome-wide analysis of transcriptional dependence and probable target sites for Abf1 and Rap1 in *Saccharomyces cerevisiae*. Nucleic Acids Res..

[40] Rhode P.R., Elsasser S., Campbell J.L. (1992). Role of multifunctional autonomously replicating sequence binding factor 1 in the initiation of DNA replication and transcriptional control in *Saccharomyces cerevisiae*. Mol. Cell. Biol..

[41] Descorps-Declère S., Saguez C., Cournac A., Marbouty M., Rolland T., Ma L., Bouchier C., Moszer I., Dujon B., Koszul R. (2015). Genome-wide replication landscape of *Candida glabrata*. BMC Biol..

[42] Shor E., Garcia-Rubio R., DeGregorio L., Perlin D.S. (2020). A Noncanonical DNA Damage Checkpoint Response in a Major Fungal Pathogen. mBio.

[43] Rivier D.H., Ekena J.L., Rine J. (1999). HMR-I is an origin of replication and a silencer in *Saccharomyces cerevisiae*. Genetics.

[44] Venditti P., Costanzo G., Negri R., Camilloni G. (1994). ABFI contributes to the chromatin organization of *Saccharomyces cerevisiae* ARS1 B-domain. Biochim. et Biophys. Acta (BBA)-Gene Struct. Expr..

[45] Lai W.K.M., Pugh W.K.M.L.B.F. (2017). Understanding nucleosome dynamics and their links to gene expression and DNA replication. Nat. Rev. Mol. Cell Biol..

