# Supplementary material for: Abf1 Is an Essential Protein That Participates in Cell Cycle Progression and Subtelomeric Silencing in Candida glabrata
Source: J Fungi (Basel). 2021 Nov 25;7(12):1005. doi: 10.3390/jof7121005 (PMC8708972; doi:10.3390/jof7121005)
Supplement: Supplementary file 1 [file jof-07-01005-s001.zip › jof-1447860-supplementary.pdf]

Supplementary tables:

Table S1 ***C. glabrata*** **and** ***E. coli*** **strains used in this study.**

E. coli **strain**	**Use**	**Genotype**	**Reference**
DH10B	Electrocompetent cells	F$^-$ *mcrA* Δ(*mrr-hsdRMS-mcrBC*) 80d*lacZ*Δ*M15* Δ*lacX74* *deoR recA1 endA1 araD139* Δ(*ara,leu*)7697 *galU galK*$^-$ *rpsL nupG*	Calvin and Hanawalt 1988
C. glabrata **strains**	**Parental**	**Genotype**	**Reference**
BG14	BG2	*ura3Δ*::Tn*903* G418^R	Cormack and Falkow 1999
abf1Δ **and** ***abf1-43***			
CGM2746	BG14	*abf1Δ::NAT*^{R} *(*pCI45 integrated*)*<br>pCI12 (pP_{ABF1}::*ABF1. URA3)*	This work
CGM3068	BG14	*abf1-43::NAT (*pCI32 integrated *BsgI)*	This work
CGM3113	CGM3068	*abf1-43::FRT*<br>Nat^S	This work
CGM3584	CGM2746	*abf1Δ::FRT*<br>pCI12 (pP_{ABF1}::*ABF1.URA3*)<br>Nat^S Ura$^+$	This work
URA3 **reporter gene integrated at** ***EPA1*** **locus**			
CGM147	BG14	*ura3Δ*::Tn*903* G418^R Tn*7* at intergenic region between *EPA1* and *EPA2* (pAP508 *Spe*I/*Bcg*I). Insertion 1 at Tel E-R	De Las Peñas *et al.* 2003
CGM148	BG14	*ura3Δ*::Tn*903* G418^R Tn*7* at intergenic region between *EPA2* and *EPA3* (pAP559 *Bsr*GI/*Sph*I). Insertion 2 at Tel	De Las Peñas *et al.* 2003

		E-R	
CGM149	BG14	*ura3Δ*::Tn*903* G418^R Tn*7* at intergenic region between *EPA3* and telomere (pAP553 *Pst*I/*Eco*RI). Insertion 3 at Tel E-R	De Las Peñas *et al.* 2003
***URA3* reporter gene integrated at *EPA4* and *EPA5* locus**			
CGM159	BG14	*ura3Δ*::Tn*903* G418^R Tn*7* at unique region between *EPA5* and *EPA4* (pAP534 *Bcg* I) Insertion 2 at Tel I-R	De Las Peñas et al, 2003
CGM160	BG14	*ura3Δ*::Tn*903* G418^R Tn*7* at intergenic region between *EPA4* and telomere (pAP471 *Apa*L I/*Xba* I)) Insertion 1 at Tel I-R	De Las Peñas et al, 2003
***URA3* reporter gene integrated at the *MTL3* locus**			
CGM454	BG14	*ura3Δ*::Tn*903* G418^R Tn7 at 643 bp downstream from alpha1 stop codon (pRZ36/*Spe*I) Insertion 4 at Tel B-L	Ramirez-Zavaleta et al. 2010
CGM458	BG14	*ura3Δ*::Tn*903* G418^R Tn7 at 166 bp upstream from alpha1 start codon, between alpha1 and alpha3 (pRZ32/*Bcg*I). Insertion 5 at Tel B-L	Ramirez-Zavaleta et al. 2010
CGM697	BG14	*ura3Δ*::Tn*903* G418^R Tn7 at 131 bp downstream from alpha3 stop codon between alpha3 and *CHAI* (pRZ40/*Bcg*I) Insertion 6 at Tel B-L	Ramirez-Zavaleta et al. 2010
***abf1-43* derivatives in *URA3* reporter strains background**			
CGM2485	CGM147	*ura3Δ*::Tn*903* G418^R Tn*7* at	This work

		intergenic region between *EPA1* and *EPA2*. *abf1-43*::*NAT* (pCI32/*BsgI* integrated)	
CGM2488	CGM148	*ura3Δ*::Tn*903* G418^R Tn7 at intergenic region between *EPA2* and *EPA3*. *abf1-43*::*NAT* (pCI32/*BsgI* integrated)	This work
CGM2491	CGM149	*ura3Δ*::Tn*903* G418^R Tn7 at intergenic region between *EPA3* and telomere. *abf1-43*::*NAT* (pCI32/*BsgI* integrated)	This work
CGM3113	CGM3068	*abf1*-43::FRT(pCI32 *Bsg*I integrated)	This work
CGM3167	CGM159	*ura3Δ::*Tn*903* G418^R Tn7 at intergenic region between *EPA5* and *EPA4*. *abf1-43::NAT* (pCI32/*BsgI* integrated)	This work
CGM3150	CGM160	*ura3Δ::*Tn*903* G418^R Tn7 at region between *EPA4* and telomere. *abf1-43::NAT* (pCI32/*BsgI* integrated)	This work
CGM3168	CGM697	*ura3Δ*::Tn*903* G418^R Tn7 at 131 bp downstream from alpha3 stop codon between alpha3 and *CHAI* (pRZ40/BcgI). *abf1-43::NAT* (pCI32/*BsgI* integrated)	This work
CGM3069	CGM458	*ura3Δ*::Tn*903* G418^R Tn7 at 166 bp upstream from alpha1 start codon, between alpha1 and alpha3 (pRZ32/BcgI). *abf1-43::NAT* (pCI32/*BsgI* integrated)	This work
CGM3180	CGM454	*ura3Δ*::Tn*903* G418^R	This work

		Tn7 at 643 bp downstream from alpha1 stop codon (pRZ36/SpeI) *abf1-43::NAT* (pCI32/*BsgI* integrated)	
CGM3840	CGM3113	*abf1*-43::FRT(pCI32 integrated *Bsgl*) pVS1(pP_{MET3}::*FLAG*-linker::*ABF1.NAT*)	This work
CGM3842	BG14	*ura3Δ::Tn903G418*^{R} pVS1(pP_{MET3}::*FLAG*-linker::*ABF1.NAT*)	This work
CGM3884	CGM3113	*abf1*-43::FRT(pCI32 integrated *Bsgl*) pRM153 (pP_{MET3}::FLAG-linker.*NAT*) Empty vector	This work
CGM3882	BG14	*ura3Δ::Tn903G418*^{R} pRM153(pP_{MET3}::FLAG-linker.*NAT*) Empty vector	This work
**pP_{ABF1}::*ABF1*, pP$_{MT1}$::*Myc::ABF1*, pP$_{MT1}$::*Flag::ABF1* and pP$_{MET3}$::*Flag::ABF1***			
CGM2391	BG14	*ura3Δ*::Tn*903* G418^R pCI12 (pP_{ABF1}::*ABF1. URA3)*	This work
CGM3123	CGM3113	*ura3Δ*::Tn*903* G418^R *abf1-43::FRT* (pCI32/ *Bsg*I integrated) pCI12 (pP_{ABF1}::*ABF1. URA3)*	This work
CGM3125	CGM3113	*ura3Δ*::Tn*903* G418^R *abf1-43::FRT* (pCI32/ *Bsg*I integrated) pGBR2.0 *URA3*	This work
CGM3453	BG14	*ura3Δ*::Tn*903* G418^R pGH8 (pP$_{MT1}$::*Myc::ABF1)*	This work
CGM3455	CGM3113	*ura3Δ*::Tn*903* G418^R *abf1-43::FRT* (pCI32/ *BsgI* integrated) pGH8 (pP$_{MT1}$::*Myc::ABF1)*	This work
CGM3588	CGM2746	*abf1Δ::FRT* NAT^R pGH8 (pP$_{MT1}$::*Myc::ABF1)*	This work
CGM3594	CGM3584	*abf1Δ::FRT* Ura$^+$ NAT^R	This work

		pCI12 (pP$_{ABF1}$::*ABF1*); pGH8 (pP$_{MT1}$::*Myc*::*ABF1)*	
CGM3838	CGM3584	*abf1*Δ::FRT Ura^{+} NAT^{R} pCI12 (pP$_{ABF1}$::*ABF1*); pPVS1 (pP$_{MET3}$::Flag-linker::*ABF1*)	This work
CGM3874	CGM3838	*abf1*Δ::FRT NAT^{R} pPVS1 (pP$_{MET3}$::Flag-linker::*ABF1*)	This work
CGM4391	BG14	*ura3Δ*::Tn*903* G418^{R} *ura*$^{+}$ pGE238 (p*P*$_{MT1}$::Flag-linker-*ABF1.URA3)*	This work
CGM4393	BG14	*ura3Δ*::Tn*903* G418^{R} Ura^{+} pGE208 (p*P*$_{MT1}$::Flag-linker-*ABF1.URA3*)	This work

Table S2 Plasmids used in this study.

Plasmid	Relevant genotype	Reference
**Cloning vectors**		
pGRB2.0	Cloning replicative vector *URA3* Ap^R pRS406::*C.g. CEN ARS*	Zordan et al. 2013
pMB11	Cloning vector with an *StuI* restriction site added Cm^R Sac^S	Lab collection
**Replicative and epitope-tagging vectors**		
pRS306	Integrative vector Amp^R *URA3*$^+$	Sikoski et al, 1989
pCN-*MET3*	*MET3*promoter, empty vector Amp^R, NAT^R	Zordan et al, 2013
pCU-*MET3*	*MET3*promoter, empty vector Amp^R, *URA3*$^+$	Zordan et al, 2013
pAP599	Cloning, integrative vector with two FRT direct repeats flanking a hygromycin resistance cassette (FRT-P_{PGK1}::*hph*::*3'*UTR_{HIS3}-FRT) for construction of multiple rounds of knock-out mutants, Amp^R, Hyg^R, *URA3*$^+$	Domergue et al, 2005
pYC44	Integrative vector FRT::*NAT*::3'UTR_{CTA1}::FRT Amp^R	Yañez-Carrillo et al, 2015
pVA59	Replicative vector c-Myc (amino terminal) pCU-*MET3*::c-Myc-linker Amp^R, *URA3*$^+$	Laboratory collection-Vidal-Aguiar unpublished
pVA78	Replicative vector pGRB2.3	Laboratory collection-Vidal-Aguiar et al, unpublished
pVA98	P_{MT1} empty vector Amp^R, *URA3*$^+$ pCU::P_{MT1}	Laboratory collection-Vidal-Aguiar et al, unpublished
pVA106	P_{MT1} empty vector Amp^R, NAT^R pCN::P_{MT1}	Laboratory collection-Vidal-Aguiar et al, unpublished
pCI32	3'UTR of *ABF1* released from pCI9 with *KpnI/XhoI*, cloned into pCI30. p*abf1-43::FRT::NAT::FRT::3'*UTR_{ABF1}	This work
pCI37	A 1.2 Kb of the 5'UTR of *ABF1*, cloned into the *Stu*I digested pMB11 vector. p*5'*UTR_{ABF1}	This work

pCI42	5'UTR of *ABF1* released with *Bam*HI/*Sac*I from pCI37, and cloned into pYC44 digested with *BamHI/SacI.*<br>p*5'UTR*$_{ABF1}$::*FRT::NAT::FRT*	This work
pCI45	*ABF1* deletion vector. 3'UTR of *ABF1* released from pCI9 with *XhoI/KpnI*, and cloned into pCI42 digested with *XhoI/KpnI.*<br>p*5'UTR*$_{ABF1}$*-FRT-NAT-FRT-3'UTR*$_{ABF1}$	This work
pGH3	A 570 bp fragment of cMyc-linker released from pVA59 with *SpeI/ClaI*, and cloned into pVA106 digested *SpeI/ClaI.*<br>pP_{MT1}::*Myc.NAT*	This work
pGH5	A 1.440 Kb PCR product of *ABF1* with *ClaI* sites added, and cloned into pMB11 digested with *StuI.*<br>p*ABF1*	This work
pGH8	A 1.44 Kb fragment of *ABF1* released from pGH5 with *ClaI*, and cloned into pGH3 digested with *ClaI*.<br>pP_{MT1}:: *Myc::ABF1.NAT*	This work
pGH9	A 2.1 Kb fragment of *hph* cassette released from pAP599 with *XbaI* and treated with T4 DNA Pol, and cloned into pMZ18 digested with *StuI/SnaBI.*<br>pP_{EPA1}::*FLP1.HPH*	This work
pVS1	A 1.44 Kb fragment of *ABF1* released from pGH5 with *Cla*I and cloned into pRM153 digested with *Cla*I.<br>pP_{MET3}::Flag::*ABF1.NAT*	This work
pGE208	A 941 bp fragment of P_{MT1} released from pRM136 with *Xba*I/*Sac*I, and cloned into pGE204 digested with *Xba*I/*Sac*I.<br>pP_{MT1}::Flag.*URA3*	This work
pGE238	A 2.024 Kb fragment of Flag::*ABF1*::T*HIS3* released from pVS with *Spe*I/*Kpn*I , and cloned into pVA98 digested with *Spe*I/*Kpn*I.<br>pP_{MT1}::Flag::*ABF1.URA3*	This work

Table S3 Oligonucleotides used in this study.

Primer (No.)	Sequence (5′-3′)	Site(s) added	Hybridization site (*ABF1*)
2580	ACTTGCACTCCGGTATCCAC	None	@1186 Fw
2579	CTCTGAACTTGGCGACATCA	None	@796 Rv
2577	CAGTCGCGTTGCCCTATTAT	None	@165 Fw
2353	TCTatcgatAAAATGGATTGACGGTATGATTTCT G	*Cla*I	@1 Fw
2354	TCTatcgatTTATTGTCCTCTTAATTCAGG	*Cla*I	@1440 Rv
1558	GCTACTGCGATTTGCCACTG	None	@-91 Fw
1559	GTTGagctcTTGTGCAGACGATCCGCAGGTCAC CG C	SacI	@385 Fw
1561	CTTctcgagGCTCCAATTATTAAAATGAATAAAA GG	*Xho*I	@+13 Fw
1562	CTTggtac**C**TTgtgcagTGCCGCCAACTTAAGCAT A	*Kpn*I, *Bsg*I	@+755 Rv
1563	TGTATTCGggtaccGCTAATTCCAG	*Kpn*I	@+933 Rv
1589	CTTGagctcGATTGTTGTGTAGGCAATATCATAG C	*Sac*I	@-1240 Fw
1590	CTTggatccCCGAACATTTGGTCAGATCACTG	*BamH*I	@+712 Rv
1834	GGGCCCGCTCCAATTATTAAAATGAATAAAG G	None	@+13 Fw
1835	CTCTGACTCCTCAATCCTTAACC	None	@+1015 Rv
1880	GTTggatcctTAGACTTCACGAGGAAGCTTGTCG TCGG	*BamH*I	@1308 Rv
1881	CTTggatccCGTTGTTTGTGTTCTCGTTGG	*BamH*I	@-1 Rv
1884	AGTGCACTTATCCTCCATCC	None	@-2134 Fw
1885	GGATCCACTAGTTCTAGAGCGGCGTTGTTTGT GTTCTCGTTGG	None	@-1 Rv
569	TACAAAGCTTGTTCACCATCGGAAGC	None	NAT resistance cassette Rv
1096	GCTTGCCTCGTCCCCG	None	NAT resistance cassette Fw
1842	CCGCTCTAGAACTAGTGGATCC	None	NAT resistance cassette Fw
1843	GGGCCCCCCCTCGAGGAC	None	NAT resistance cassette Rv

*The restriction sites added to the primers are indicated in small case, blue letters.

Table S4. Duplication times of strains containing *ABF1* driven by the *MET3* promoter under expressing or repressing conditions.

Promoter	Strain	Duplication time (min ± SD)				
		Rich		OFF		ON
		YPD	SC + CAA[a]	YNB + 2 mM met + cys	YNB + 0.2 mM met +cys	YNB
Empty vector	WT (*ABF1*$^{+}$)	54 ± 2	67 ± 2	194 ± 3	149 ± 25	112 ± 4
	abf1-43	67 ± 16	73 ± 4	211 ± 18	179 ± 35	127 ± 8
P_{ABF1}[b]	*abf1Δ*/p*ABF1*	56 ± 1	59 ± 0	118 ± 5	98 ± 0	76 ± 0
P_{MET3}[c]	WT/pFlag-*ABF1*	55 ± 1	69 ±2	194 ± 6	162 ± 38	138 ± 9
	abf1-43 / Flag-*ABF1*	62 ± 2	74 ± 8	200 ± 9	150 ± 29	142 ± 9
	abf1Δ/ pFlag-*ABF1*	0	0	0	0	127 ± 4

[a] SC medium is YNB supplemented with ammonium sulfate, 0.2% glucose and 0.6% casaminoacids

[b] Plasmid containing *ABF1* driven by its own promoter

[c] Plasmid containing cMyc-*ABF1* driven by the repressible promoter of the *MET3* gene. The *MET3* promoter is only expressed in the absence of both met and cys (YNB, ON medium).

Numbers represent the mean of at least 3 independent experiments.

Table S5: Cell viability of *abf1Δ*/pP_{MET3}::Flag-*ABF1* strains under repression of *ABF1*

Time (h)	CFU[a]	
	MEDIA[b]	
	ON	OFF
**Experiment 1[c]**		
0	1.04×10^6	9.73×10^5
1	8.37×10^5	9.97×10^5
2	1.71×10^6	1.64×10^6
3	2.36×10^6	1.90×10^6
4	3.42×10^6	2.27×10^6
5	4.68×10^6	2.40×10^6
6	7.22×10^6	2.75×10^6
24	9.66×10^7	5.33×10^4
**Experiment 2[d]**		
0	2.27×10^6	1.10×10^6
7	3.51×10^7	3.70×10^6
8	4.18×10^7	2.37×10^6
9	4.08×10^7	1.23×10^6
10	1.06×10^8	6.67×10^5
11	1.18×10^8	4.67×10^5
12	1.13×10^8	5.33×10^5
24	1.58×10^8	1.33×10^5

[a] CFU: Colony forming units per mL of culture

[b] Saturated cultures of the *abf1Δ*/pP_{MET3}::Flag-*ABF1* strain were diluted to an OD_{600} of 0.1 into either ON media (YNB without amino acids) or OFF media (YNB + 0.2 mM met and cys) and incubated at 30°C with shaking. Samples were taken at the indicated time points, diluted and plated on YNB (ON). Colonies were counted and total colony forming units (CFU) were calculated.

[c] Experiment 1: Samples from cultures of either ON or OFF media were taken every hr during the first 6 h, diluted and plated on YNB (ON) plates. Colonies were counted and total colony forming units (CFU) were calculated.

[d] Experiment 2. Cells inoculated into either ON or OFF media were incubated for the first 6 hr after which samples were taken every hour (starting at 7 h after the cells were initially diluted into each media) and diluted and plated as described in c. All experiments were performed three times. Data from one representative experiment are shown.

Table S6. Percentage of unbudded and budded cells under Flag-*ABF1* repression and Myc-*ABF1* overexpression.

Strain[a]	Plasmid[a]	Effect	Medium[b]	Time[c] 6 h UB (%)	6 h B (%)	9 h UB (%)	9 h B (%)
abf1Δ	pP$_{MET3}$::Flag-*ABF1*	*ABF1* repression	ON	66.3	34.6	81.4	18.5
			OFF	52.6	47.3	42.5	57.5
	pP$_{MT1}$::Myc-*ABF1*	*ABF1* O-E	- Cu	64.8	35.1	66.2	33.7
			+ Cu	34.6	65.3	55.3	44.6

[a] *C. glabrata* null strain *abf1Δ* containing the shut off plasmid pP$_{MET3}$::Flag-*ABF1* or the over expressing plasmid pP$_{MT1}$::Myc-*ABF1*.

[b] ON medium is minimal medium YNB where the *MET3* is active. OFF is YNB minimal medium with 0.2 mM each of methionine and cysteine where the *MET3* promoter is repressed. – Cu is YNB minimal medium and + Cu is YNB minimal medium with 50 μM $CuSO_4$ to induce the *MT-1* promoter.

[c] Time at which samples were taken after diluting stationary phase cells of each strain into the indicated medium. Cell suspensions were viewed and counted under the microscope and the percentage of unbudded (UB) or budded (B) cells was calculated. Budded cells include both, small and large budded.

Supplementary Figures

Supplementary Figure S1

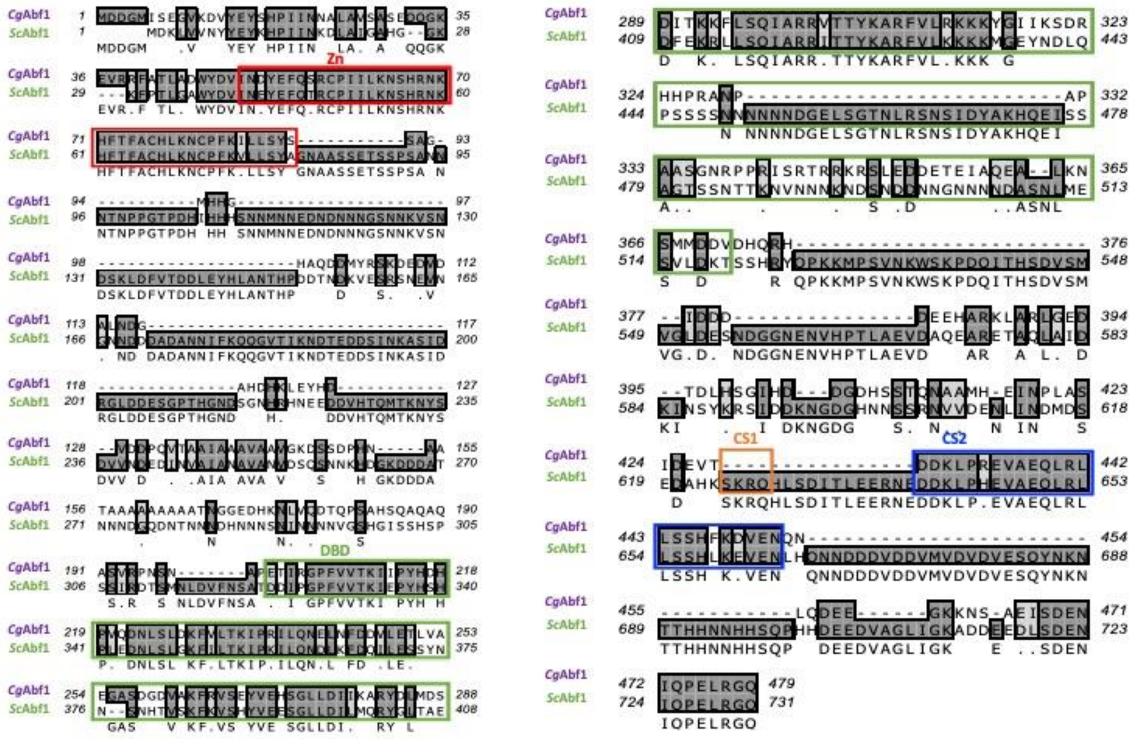

Figure S1: CgAbf1 is similar to its ortholog ScAbf1 and conserves the functional domains. The genome of *Candida glabrata* contains the *ABF1* gene encoding the CgAbf1, which conserves the N-terminal atypical Zn-finger domain (indicated with Zn and a red rectangle), followed by a second DNA-binding domain in the middle section of the protein, indicated as DBD with a green rectangle. At the C-terminal end the CS2 domain is well conserved (indicated as CS2 and in blue rectangle).

Supplementary Figure S2

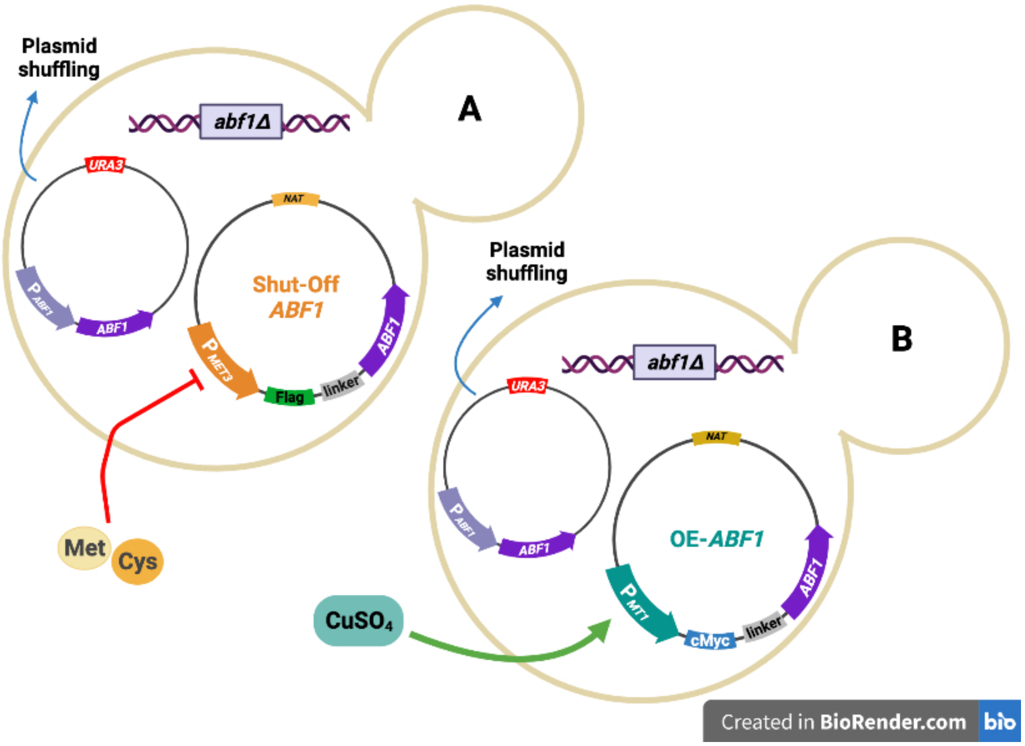

Figure S2: Plasmid shuffling scheme to generate a "shut off *ABF1* system" or an "*ABF1* over expression system" (OE-*ABF1*) in the *abf1Δ* background
Schematic representation of the plasmid shuffling strategy followed to generate the *abf1Δ*/pP$_{MET3}$::Flag-*ABF1* strain (shut off system) and the *abf1Δ*/pP$_{MT\text{-}1}$::cMyc-*ABF1* strain (OE system). The *abf1Δ* strain complemented with the plasmid containing the *ABF1* wild-type gene under its own promoter was transformed with either the shut off or the OE plasmid. Cells of each strain were grown under non-selective conditions to allow them to lose the wild-type *ABF1* plasmid, generating either the shut off strain (*abf1Δ*/pP$_{MET3}$::Flag-*ABF1*) or the over-expressing strain (*abf1Δ*/pP$_{MT\text{-}1}$::cMyc-*ABF1*). Addition of met and cys to the shut off strain leads to complete repression of *ABF1*, while addition of $CuSO_4$ to the overexpressing strain results in induction and overexpression of *ABF1* as indicated.

Supplementary Figure S3

A

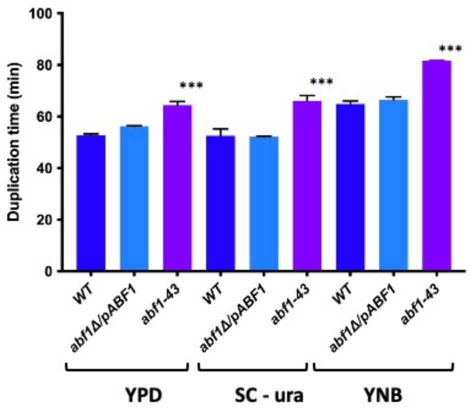

Strain	Duplication time (min)[a]		
	YPD	SC - ura	SC
Parental	52.7±0.6	52.0±2.7	64.9±1.1
abf1Δ/pP$_{ABF1}$::ABF1.URA3	56.2±0.1	52.1±0.2	66.5±1.0
abf1-43	64.4±1.3	66.1±2.0	81.6±0.1

B

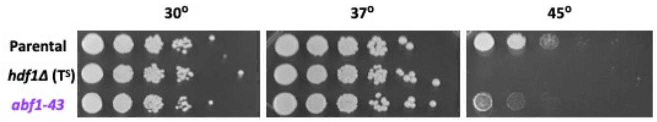

Figure S3: *abf1-43* mutant has a longer duplication time and is temperature sensitive at 45°C. A) Growth rate of the parental strain (*ABF1*), the *abf1Δ* strain complemented with the plasmid containing *ABF1* driven by its own promoter and the *abf1-43* mutant strain in different media: rich (YPD); SC – ura: minimal media supplemented with casamino acids (0.6%), 2% glucose and 0.5% ammonium sulfate; SC: minimal media with 2% glucose and 0.5% ammonium sulfate. On the right side, the table with the duplication times are shown for each strain in each medium.

[a] Values correspond to the mean of three biological assays. B) Stationary phase cells from the indicated strains were adjusted to an OD_{600} of 1 and 5 μL of serial 10-fold dilutions were spotted on to YPD and incubated at the indicated temperatures. The *hdf1Δ* strain is a control of a temperature-sensitive strain unable to grow at 45°C.

Supplementary Figure S4

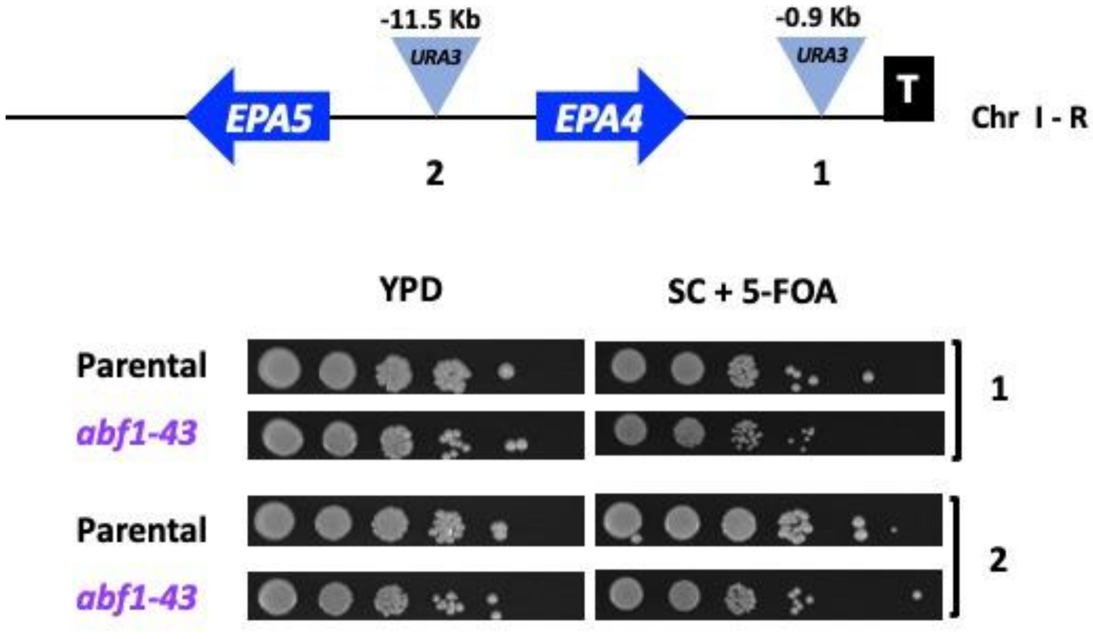

Figure S4: *Cg*Abf1 is not required for silencing at positions very close to some telomeres. Top: Schematic representation of the subtelomeric region of the Chr I-R in *C. glabrata*. The light blue triangles represent the position of two independent insertions of the *URA3* reporter introduced either in the wild-type parental strain or in the *abf1-43* truncation mutant. The distance from the telomere is indicated above each blue triangle, and the numbers beneath the triangles correspond to the insertions carried by each strain in the plate growth assay below. Ten-fold dilutions of cells grown to stationary phase were spotted onto the media indicated, incubated at 30 °C for 48 h and photographed.
